# Atomic-Scale View at the Segregation of Alkali Metals toward the KTaO_3_(001) Perovskite Surface

**DOI:** 10.1021/acsami.4c13795

**Published:** 2024-12-10

**Authors:** Aji Alexander, Michele Reticcioli, Llorenç Albons, Jesús Redondo, Marco Corrias, Igor Píš, Zhichang Wang, Viktor Johánek, Josef Mysliveček, Cesare Franchini, Dominik Wrana, Martin Setvin

**Affiliations:** † Department of Surface and Plasma Science, Charles University, Prague 18000, Czech Republic; ‡ Faculty of Physics and Center for Computational Materials Science, University of Vienna, Sensengasse 8/12, Vienna 1090, Austria; § Department of Polymers and Advanced Materials, Centro de Física de Materiales, University of the Basque Country UPV/EHU, San Sebastián 20018, Spain; ∥ CNR - Istituto Officina dei Materiali (IOM), Trieste 34149, Italy; ⊥ International Center for Quantum Materials, School of Physics, Peking University, Beijing 100871, China; # Dipartimento di Fisica e Astronomia, Università di Bologna, Bologna 40126, Italy; ∇ Marian Smoluchowski Institute of Physics, Jagiellonian University, Krakow 30-348, Poland

**Keywords:** cation segregation, KTaO_3_, scanning probe microscopy, perovskites, solid oxide fuel cells, ORR, photoelectron spectroscopy, density functional theory

## Abstract

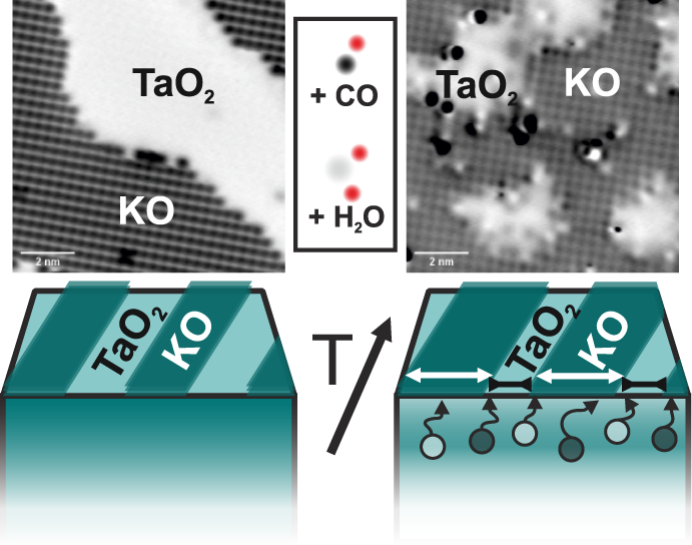

Perovskites exhibit outstanding performance in applications such as photocatalysis, electrochemistry, or photovoltaics, yet their practical use is hindered by the instability of these materials under operating conditions, specifically caused by the segregation of alkali cations toward the surface. The problem arises from the bulk strain related to different cation sizes, as well as the inherent electrostatic instability of perovskite surfaces. Here, we focus on atomistic details of the surface-driven process of interlayer switching of alkali atoms at the inorganic perovskite surface. We show that the (001) surface of KTaO_3_ cleaved at room temperature contains equally populated TaO_2_ and KO terminations, while the uncompensated polarity of these terminations promotes diffusion of KO from the subsurface toward the topmost surface layer at temperatures as low as 200 °C. This effect is directly probed at the atomic scale by Atomic Force Microscopy and the chemical properties of the resulting surfaces are investigated by the adsorption of CO and H_2_O. The experiments indicate that KO segregation is associated with the formation of K and O vacancies in the near-surface region, which is further supported by depth-dependent X-ray Photoelectron Spectroscopy measurements and Density Functional Theory calculations. Our study shows that the KO segregation influences the surface reactivity both toward CO and water, which was probed at the atomic scale.

## Introduction

Perovskite oxides are appealing catalysts for electrochemical anodic reactions, such as oxygen evolution (OER) and methanol oxidation (MOR), as well as cathodic reactions, such as hydrogen evolution (HER) and CO_2_ reduction (CO2RR).^1^ They are also widely used in solid oxide fuel cells (SOFCs) for oxygen reduction reactions (ORR).^2^ Recently, tantalate and niobate perovskites have attracted increasing attention due to the catalytic properties of their respective transition metals,^3,4^ their highly efficient photocatalytic performance,^5−7^ as well as their tunable ferroelectric properties.^8^ Potassium tantalate, KTaO_3_ (KTO), is an incipient ferroelectric material with a theoretical Curie temperature T_C_ below 0 K.^9^ The T_C_ can be tuned by niobium doping, achieving bulk T_C_ up to 700 K,^10^ which makes this material attractive for ferroelectric (pyro-, piezo-) catalysis.^11,12^ Here, we investigate one of the main challenges in the application of perovskites, that is, the instability of their surfaces.^13−15^ This instability is acute in polar terminations, which require compensating the surface polarity^15^ and fulfilling the Tasker’s criteria.^16^

The polar (001) plane of KTaO_3_ is used here as a prototype of a broader perovskite family with cation oxidation states +1/+5, including important photocatalytic materials such as LiNbO_3_, LiTaO_3_, NaNbO_3_, or NaTaO_3_, holding record-high efficiencies toward water splitting.^6^ The initial surface is prepared by cleaving at room temperature along the (001) plane,^14^ resulting in a bulk-terminated surface with equally populated KO and TaO_2_ terminations. These terminations have uncompensated polarity: the KO domain holds a formal ionic charge of −1*e* per unit cell, while TaO_2_ has an opposite charge. Such polar surfaces are inherently unstable^14^ and seek ways to compensate for this polarity, such as electronic reconstructions, structural reconstructions, ferroelectric relaxations, or the incorporation of external species.^15,17,18^

Another important route for polarity compensation is the segregation of cations toward the surface.^19,20^ Cation migration under operating conditions is a major drawback in the application of perovskites at the industrial level. It has been identified to play a significant role in the loss of efficiency of perovskite-based solar cells^21,22^ and to cause anode/cathode loss of conductivity and poisoning in fuel cells.^2^ The particular challenge posed to the use of perovskite oxides as cathodes in solid oxide fuel cells (SOFCs) comes from the degradation caused by the high-temperature annealing, deteriorating the electrochemical activity, especially for oxygen reduction reaction (ORR).^23^ In the case of reduction reactions like HER, the activity of perovskites is driven by surface enrichment of BO_*x*_ phases and subsequent reduction to form B metal-rich reconstructions or clusters.^1^ The process is initiated by A-site segregation and leaching, in which the initial stage is probed in this study.

In the case of ABO_3_ perovskites, the segregation of A cations and the formation of stable AO/BO/ABO_3_ secondary phases have been reported to occur in both reducing and oxidizing environments by a bunch of experimental techniques (XPS, XRD, AFM, SIMS, and LEIS) on a number of compounds (KTaO_3_, KNbO_3_, NaTaO_3_, NaNbO_3_, LiNbO_3_, BaTiO_3_, SrTiO_3_, and PbBiO_3_),^24−28^ or complex mixed perovskites and heterostructures (LaAlO_3_/SrTiO_3_, NdGaO_3_/SrTiO_3_, La_1–*x*_Sr_*x*_MnO_3_ (LSM), or La_0.6_Sr_0.4_Co_0.2_Fe_0.8_O_3−δ_ (LSCF)).^18,29−31^ Similar to our study, an oxygen vacancy formation has been indicated as the prerequisite for the secondary phase growth.^31^ Also, many attempts have been made to propose a theoretical model for the formation of new phases and ways to avoid them e.g., by using cations of different sizes^19,32^ or strain engineering.^20^ However, an atomic-level description of the initial stages of cation segregation and surface interactions with relevant molecules is still lacking.

In this work, we provide an atomically resolved view of the chemically important polarity compensation mechanism in KTaO_3_. Unlike other approaches, we start from the as-cleaved bulk reconstructed surfaces and investigate initial stages of the A-type cation segregation. The migration of KO from the subsurface regions toward the topmost surface layer^27,28,33^ results in an increased coverage of KO in the detriment of TaO_2_ domains. This process starts at temperatures as low as 300 °C, which is well below the typical operation temperature of SOFCs, which lies in a range of 700–1000 °C, rarely reaching temperatures below 600 °C.^34^ We show that the evolution of the surface layer confers distinct changes in chemical reactivity toward H_2_O and CO adsorption; these probe molecules were tested due to their relevance for the photocatalytic water splitting and CO_2_ reduction. We correlate the experimental observation of morphological changes occurring during annealing with area-averaging analytical methods: an UHV-cleaved KTaO_3_ (001) surface is examined using synchrotron-based X-ray photoelectron spectroscopy (XPS) and noncontact atomic force microscopy (nc-AFM). Density Functional Theory (DFT) is used to discuss the atomic configurations and energy balance of the involved processes.

## Experimental Section

### Experimental Methods

KTaO_3_ crystals from various sources and various doping levels have been investigated. Undoped (insulating) material purchased from Stanford Advanced Materials or *n*-doped (conducting) crystals were prepared by solidification from a nonstoichiometric melt in the Oak Ridge National Laboratory. The *n*-type doping was achieved by including trace concentrations (<1%) of rare earth elements (Sr, Ca, or Ba). The results presented in this work were independent of the crystal source and doping levels.

Surface morphology was investigated in a double-vessel UHV system equipped with a commercial cryogenic STM/AFM head (Scienta Omicron, Polar). The base pressure was 1 × 10^–10^ mbar. The KTaO_3_ bulk single crystals were first outgassed in UHV at temperatures between 500 and 650 °C and subsequently cleaved at room temperature by a tungsten carbide blade of a UHV mechanical cleaver.^35^ This procedure results in smooth areas of bulk-terminated surface, spanning from micrometers to hundreds of micrometers, separated by multisteps of tens to hundreds nanometers high; more details are provided in Figure S8.

Sample annealing was performed in a manipulator equipped with a BN heater; the sample was kept at the specified temperature for 15 min in each annealing cycle. The annealing and cooling rates were approximately 30 K/min. Oxygen partial pressure during the annealing in ultrahigh vacuum is estimated to be below 10^–13^ mbar.^36^

Tuning-fork-based AFM sensors with a separate wire for the tunneling current were used (*k* = 1900 N/m, *f*_0_ = 32 kHz, *Q* ≃ 20,000), and the deflection signal was measured by a differential cryogenic preamplifier.^37^ Tungsten (W) tips were electrochemically etched with a 2 M NaOH water solution and thoroughly cleansed in hot water before being glued to the tuning fork of the sensor. The apex of the tip was treated on the clean sputtered-annealed Cu (110) to ensure sharp stable metallic character. The AFM images shown in this article were taken at both constant frequency and constant height modes.

The clean (1 × 1) surface of KTaO_3_(001) obtained after UHV cleaving was immediately dosed with deionized water from the ultrapure liquid water reservoir attached to the UHV preparation chamber to compensate for the electrostatic instability of the (001) surface. The dosage rate of the water is approximately 170 L per 100 min at a pressure of 3.8 × 10^–8^ mbar. The experiment was then set to have annealing cycles from 100 to 620 °C with steps of approximately 100 °C for 15 min each. The quoted temperatures were estimated from the readout of a thermocouple attached to the sample stage, pyrometer measurements, and calibration of the sample annealing power (all three methods were used simultaneously to guarantee equal temperature calibration in different UHV systems). The absolute error of the temperature measurement was estimated to be ±20 °C.

The TPD measurements were carried out in a separate UHV system with a base pressure of 5 × 10^–11^ mbar. The sample was cooled by a Janis ST-400 UHV liquid-He flow cryostat and heated by direct current through the backplate. Isotopically labeled ^13^CO was dosed by an effusive molecular beam with a hat-shape profile^38,39^ and a linear temperature ramp of 0.2 K/s was used. This slow ramp speed effectively minimizes the temperature gradient inside the sample and allows an accurate measurement of the temperature. The TPD flux data were detected and acquired by a HIDEN quadrupole mass spectrometer, which had a line-of-sight configuration. Detailed descriptions of similar TPD measurements can be found elsewhere.^40^

The comprehensive elemental and chemical characterization of the sample was carried out using synchrotron X-ray photoelectron spectroscopy at the BACH beamline of the CNR at the Elettra synchrotron facility (Trieste, Italy). The system offers an ultrahigh vacuum (UHV) with a base pressure of 4 × 10^–10^ mbar and is equipped with a mechanical cleaver and a Scienta R3000 hemispherical analyzer positioned at a 60° angle from the incident beam direction. The X-rays were linearly polarized with the polarization vector parallel to the scattering plane. Photoemission data were collected in a normal emission geometry at a takeoff angle of 90°. The total instrumental energy resolution was set to 0.2 and 0.4 eV for photon energies of 650 and 915 eV, respectively. Sample annealing was performed by using a manipulator with a PBN ceramic heater. The sample was annealed for 15 min per cycle and laterally shifted to avoid regions potentially influenced by the synchrotron beam during previous measurements.

Apart from the synchrotron source, UHV lab nonmonochromatic X-ray source (base pressure <10^–9^ mbar) operated with Al anode (*h*ν = 1486.4 eV) was also employed to trace the sub-surface-related phenomena. This system is equipped with a SPECS Phoibos MDC 9 electron energy analyzer configured for normal and grazing detection angles. Apart from a wide survey spectrum, spectral regions of K 2p, Ta 4f, and C 1s were acquired. The KolXPD software^41^ was used for data fitting after Shirley’s background subtraction in all cases. The O 1s components were fitted with Voigt functions, while the K 2p (split: 2.76) and Ta 4f (split: 1.89) spectra were fitted with Voigt doublets functions.

### Computational Methods

Density functional theory nonspin-polarized calculations were performed by using the Vienna ab initio simulation package (VASP).^42−44^ We adopted the strongly constrained and appropriately normed meta-generalized gradient approximation (SCAN),^45^ with the inclusion of an on-site effective U of 4.0 eV on the d orbitals of Ta atoms.^46,47^ We used a plane-wave energy cutoff of 500 eV, and a 3 × 3 × 1 grid for the sampling of the reciprocal space.

We modeled the defect-free “labyrinth” KTaO_3_(110) surface by constructing slabs with 9 and 10 TaO_2_ and KO layers, respectively. The terminating KO layer on both sides of the slab covers only half of the underlying TaO_2_ terrace, as in the defect-free labyrinth. A vacuum region of approximately 40 Å was used. We constructed slabs with different sizes (√2 × *n*√2 R45°, with *n* = 4, 5, 6, 7) to investigate the properties of the labyrinth phase with KO/TaO_2_ stripes at different lateral width. K and O vacancies were modeled to study the segregated phase. An accurate description of all configurations analyzed is included in section 9 of the Supporting Information.

All atomic coordinates (except atoms on the central TaO_2_ layer) were relaxed using standard convergence criteria (residual forces smaller than 0.02 eV/Å). The lattice constant was kept at an optimized bulk value of 4.03 Å. Graphical representations of the models are shown in the Supporting Information, and all structure files are provided therein. All graphical representations were produced by using VESTA.^48^

The on-site average electrostatic potential was calculated using probing particle radii of 0.7215, 1.3383, and 1.2538 Å for O, K, and Ta atoms, respectively. The Δ*V* value reported in the figures is referenced to the mean value of the corresponding data points.

### Atom Counting Algorithm

To count the atoms of the images shown in this work (Figure S1), a template-matching procedure based on the open-source AiSurf^49^ Python package was performed. This procedure is now included in AiSurf.

The first step consists of obtaining a template that matches the feature we want to count, in our case atoms. To do so, we exploit the basic routine of AiSurf, based on SIFT^50^ and clustering algorithms.^51^ Once the cluster containing the desired features has been found, each cluster element is automatically cropped around its center and the template is obtained by calculating their median.

**Figure 1:**
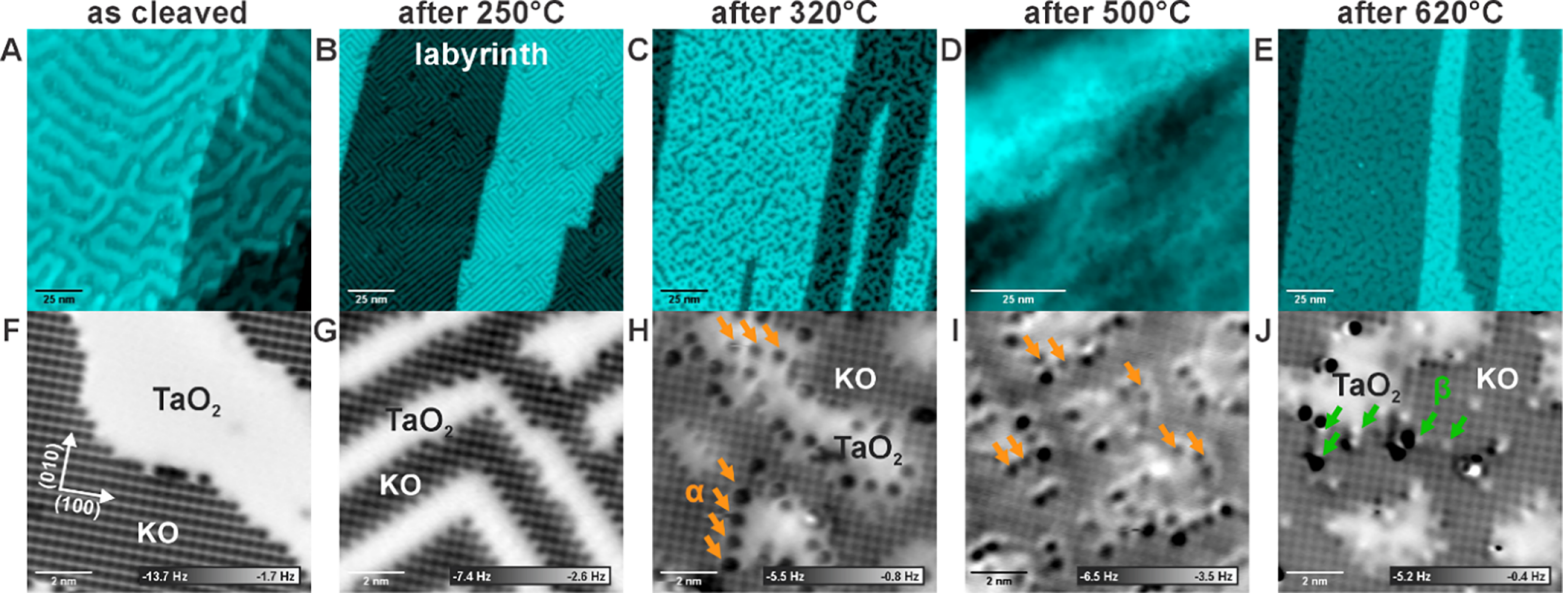
Evolution of the KTaO_3_(001) surface with annealing under UHV conditions. Panels A–E show overview AFM images of the surface morphology (measured in the constant frequency shift mode); panels F–J show atomically resolved constant-height AFM images. (A, F) Surfaces cleaved at room temperature. (B, G) After additional annealing to 250 °C. (C, H) Annealed to 320 °C; arrows indicate α-type point defects). (D, I) Annealed to 500 °C; arrows indicate α-type point defects. (E, J) annealing to 620 °C, arrows indicating β-type point defects.

A correlation map between the image and the template is obtained with a template-matching function in the scikit-image^52^ package. The peaks of the correlation map are associated with the locations of the desired features and are counted with a function of scikit-learn. This method provides accurate estimations given a proper template and parameter selection (described in the manual). As shown in Figure 1E, false detections are mainly located where the topography of the image suddenly changes (e.g., due to the presence of defects, between terminations, or image artifacts).

## Results and Discussion

### Surface Structure and Evolution

The evolution of as-cleaved KTaO_3_(001) surfaces upon thermal annealing under an ultrahigh vacuum is summarized in Figure 1. Panels A–D show overview images of the surface morphology. The as-cleaved (1 × 1) surface (Figure 1A) consists of alternating stripes of KO and TaO_2_ terminations, where the KO always protrudes from the surface. This peculiar morphology is caused by the polarity compensation mechanism trying to limit the area of charged KO^+^ or TaO_2_^–^ surfaces.^14^ The typical width of the KO/TaO_2_ stripes is 3–8 nm. Additional information on the large-scale cleaved plane behavior and the assignment of the KO and TaO_2_ terminations is provided in Figure S8. Annealing the surface to temperatures above 250 °C results in the reorganization of the terrace shape into a structure referred to as the “labyrinth” (see Figure 1B). Here, all the step edges are oriented along the <110> directions, which corresponds to a nonpolar step termination. The terraces adopt a characteristic width of 1.3 ± 0.3 nm, which is given by a balance between the polarity of the bulk-terminated surface and the formation energy of a step.^14^ Annealing to temperatures above 300 °C results in further rearrangement of the terraces and step orientations (see Figure 1C–E).

Details of the surface structure are provided in atomically resolved AFM images (constant height) in Figure 1F,G which were obtained after cleavage and gradual annealing of the sample. In the whole temperature range, the bulk-terminated (1 × 1) structure is preserved. Notably, the strong preference for the (1 × 1) surface termination is characteristic of KTaO_3_(001) and has not been observed for other perovskites.^53^ At temperatures below 300 °C, the surface stabilizes itself mainly through the rearrangement of the KO terraces. At temperatures above 300 °C, new mechanisms come into play. Most notably, the TaO_2_ regions develop numerous point defects, see the dark spots marked “α” in Figure 1H,I. Above 620 °C, the KO terraces start to develop extended in-plane defects, marked as “β” (bright lines) in Figure 1J. In the early works,^14^ these two types of defects were tentatively attributed to the segregation of impurities. However, we have repeatedly observed the same defects on numerous samples from different vendors and with different doping levels (see the Methods section) and no traces of unwanted extrinsic impurities have been observed in XPS measurements (see Figure S4 for overview).

**Figure 2:**
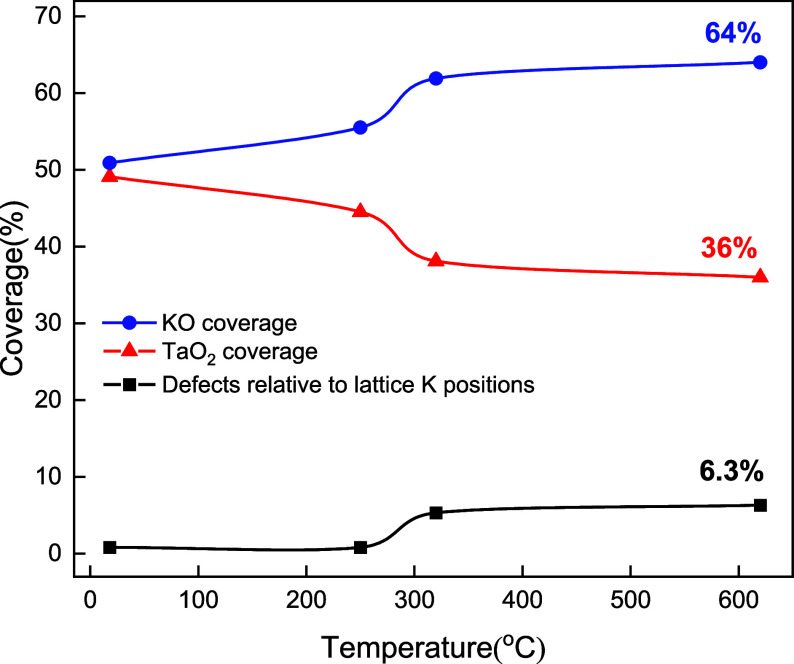
Temperature evolution of the KO/TaO_2_ ratio at the KTaO_3_(001) surface. The coverage of KO (blue dots) and TaO_2_ (red triangles) terminations calculated from AFM images. An increase in KO coverage after annealing to 250 °C is accompanied by the formation of defects in the surface layer (black squares). After annealing to 620 °C, KO coverage increases to 64%, whereas TaO_2_ shrinks to 36% of the KTaO_3_(001) – (1 × 1) area.

Another mechanism for surface stabilization, not immediately apparent from the images, plays a significant role—specifically, the interlayer switching of potassium oxide between the topmost surface layer and the near-surface region. Careful analysis of the thermally annealed surfaces shows that the area of KO gradually increases at the expense of the TaO_2_ regions. Figure 2 shows the areas of KO derived from atomically resolved AFM images, evaluated by counting the surface K atoms (see Figure S1 for details). While the as-cleaved surfaces fulfill the expected KO/TaO_2_ ratio of 50:50%, annealing to temperatures as low as 200 °C already leads to a 55:45 ratio (in the corresponding “labyrinth” structures). After annealing above 300 °C, the coverage of KO converges toward ∼65%, while the remaining ∼35% of TaO_2_ regions host a significant number of additional defects.

**Figure 3:**
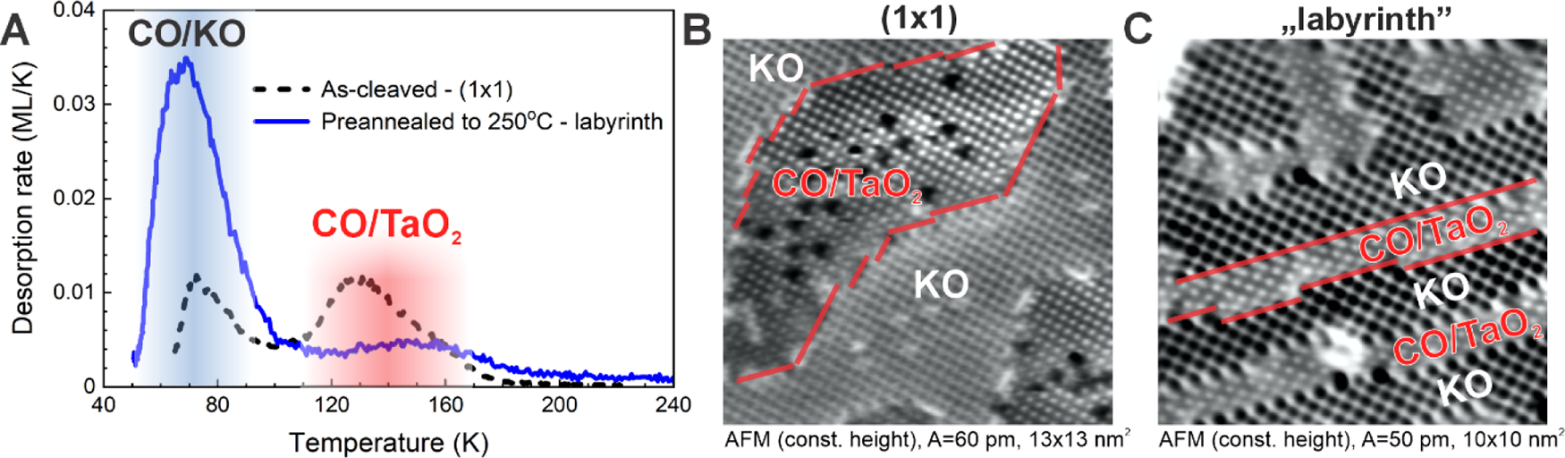
Probing the surface structure by CO adsorption. (A) Saturation-coverage TPD spectra of ^13^CO from as-cleaved KTaO_3_(001) surface (1 × 1 surface) and the surface annealed to 250 °C (labyrinth structure). (B) Constant height AFM image of a saturation coverage of CO dosed at *T* = 100 K. (C) Constant height AFM image of the labyrinth structure with a saturation coverage of CO dosed at *T* = 80 K. At this temperature, CO adsorbs at the TaO_2_ termination only. Red lines mark the border between the KO and TaO_2_ terraces.

**Figure 4:**
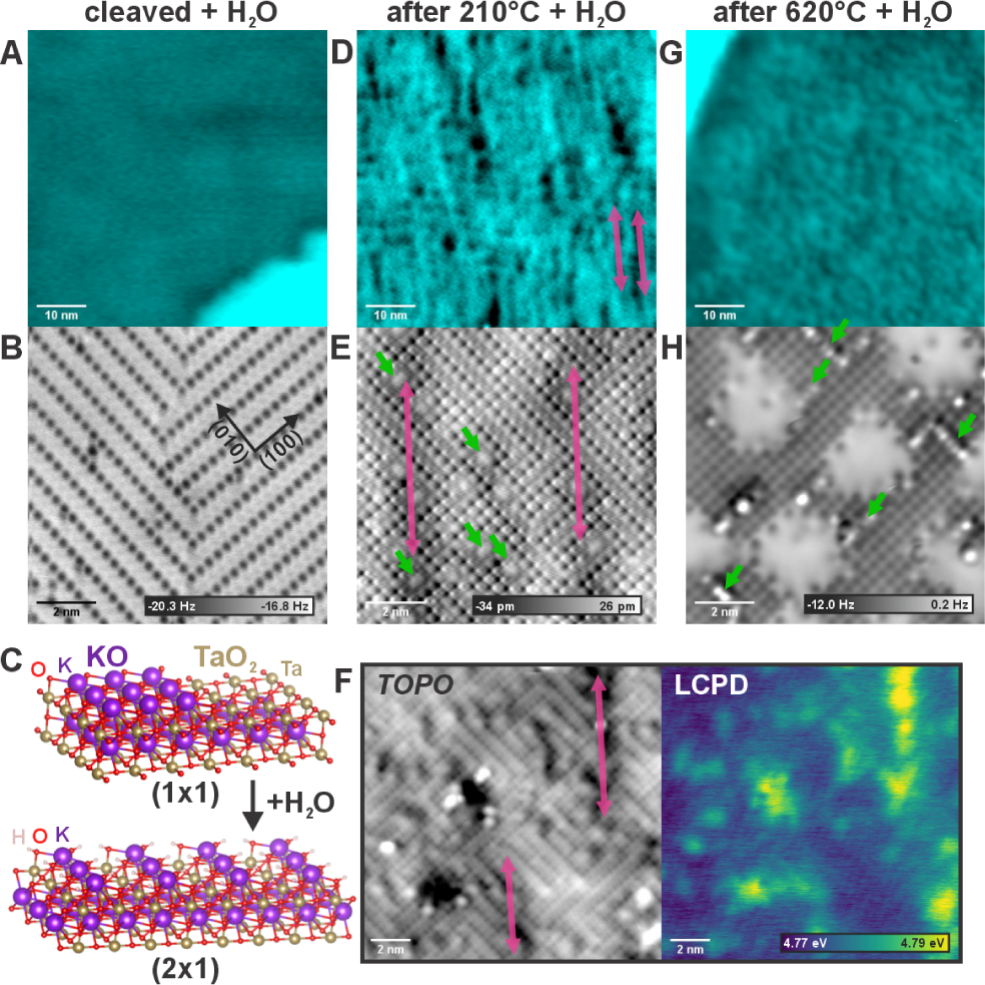
Water interaction with differently treated surfaces. (A, B) As-cleaved surface after exposure to 170 L of H2O gas at room temperature, showing a homogeneous (2 × 1) reconstruction. (C) Atomic model of the as-cleaved (1 × 1) and water-terminated surface (2 × 1). D, E) The “labyrinth” structure (as in Figure 1B,G) was first created by cleaving and annealing the sample to 210 °C in UHV. Subsequent exposing of the surface to 170 L H_2_O at room temperature results in a surface shown in panels D and E. (G, H) Surface annealed to 620 °C in UHV and subsequently exposed to 170 L of water vapor at RT. (F) KPFM maps (topography and calibrated LCPD) of the water-terminated surface created at the surface preannealed to 210 °C.

To support the observation of KO switching and to learn further details about the involved surface reactivity, we investigated the interaction of the annealed surfaces with gas probe molecules: carbon monoxide (Figure 3) and water vapor (Figure 4). In Figure 3, we use the specific adsorption properties of CO to verify the shrinkage of the TaO_2_ regions and expansion of the KO regions. CO preferentially adsorbs on the TaO_2_ surface at low temperatures, since interaction is stronger than on KO-terminated KTaO_3_(001).^40^ In the collected temperature-programmed desorption (TPD) spectra in Figure 3A, the lower-temperature peak at ∼75 K corresponds to CO desorption from KO and the broader peak spanning from 100 to 180 K originates from CO adsorbed on TaO_2_. The desorption rates are calibrated to absolute values, but the KO peak is not fully captured because the lowest temperature achievable in the experimental setup already lies within this peak. Figure 3B shows an atomically resolved AFM image of the as-cleaved surface exposed to a saturation coverage of CO at *T* = 100 K. At this temperature, the CO only sticks to the TaO_2_ terraces, binding to surface Ta atoms.^40^ This is clearly visible in the regions marked with CO/TaO_2_. Here, the TaO_2_ terraces filled with CO occupy exactly 50% of the surface.

The TPD spectrum of CO dosed at the labyrinth structure produced by annealing is shown in Figure 3A. The desorption peak from the TaO_2_ terraces is significantly smaller with its area approximately halved as compared to the as-cleaved surface. The desorption peak from KO is much higher, yet quantitative comparison is difficult because the lowest achievable temperature overlaps with this peak and was different in the respective experiments. A clear verification of the decreasing number of surface Ta sites is shown in the AFM image in Figure 3C. Here, the number of CO molecules (corresponding to the area of TaO_2_ terraces) becomes significantly lower than 50%; only ≈1/3 of the surface area is covered by CO molecules. In some regions of Figure 3C, the borders between the KO and TaO_2_ regions are marked by red lines to highlight the disproportion of the surface areas corresponding to KO and CO/TaO_2_.

Microscopic images in Figure 4 show the interaction of the KTaO_3_(001) surface with water vapor at room temperature. Here, we focus on the role of sample preannealing (as in Figure 1) on the surface chemistry. Images of the as-cleaved surfaces exposed to 170 Langmuir of water are shown in Figure 4A,B. Here, the water dissolves the KO islands and disperses the material homogeneously across the surface, resulting in a well-defined (2 × 1) reconstruction of potassium oxyhydroxide^14^ (see Figure 4A–C). The same experiment was applied on surfaces that were preannealed to various temperatures. Figure 4D,E shows the result after exposing the labyrinth structure to 170 L water. The atomically resolved image (Figure 4E) shows that the resulting surface is indeed covered by a well-developed (2 × 1) reconstruction. However, there are additional features that are visible in the image. First, there are regions with locally darker (indicated by arrows in Figure 4E) and brighter background contrast, with the periodicity corresponding to the original labyrinth structure. This observation is also well pronounced in the overview AFM image in Figure 4D. This modulation likely originates from long-range forces (mostly van der Waals and electrostatic), where a darker contrast corresponds to regions that locally contain a higher concentration of atoms either at the surface or subsurface. In addition to this long-range modulation, the surface in Figure 4E contains a new type of point defect (marked in green). The characterization of this labyrinth system is complemented by a Kelvin Probe Force Microscopy (KPFM) map shown in Figure 4F. Surface defects carry a different charge state, inducing a slight variation in the local effective work function.

Surfaces preannealed to temperatures above 300 °C do not form the (2 × 1) reconstruction after exposure to water, but the surface retains a well-defined (1 × 1) termination, see Figure 4G,H. The extended defects in panel H appear the same as the β-defects in Figure 1. This indicates that the surface polarity is better compensated than in the case of the labyrinth structure, and additional water molecules do not provide enough thermodynamic driving force toward the transition into the (2 × 1) hydroxylated structure.

A graphical illustration of all experimental pathways can be found in the supplement (Figure S2). Surface investigations presented above provide convincing evidence that vacuum annealing of the KTaO_3_(001) surface leads to an increase in the KO area. The increased areas of surface KO (Figures 2, 3, and S1) raise the question about the source of the excess material. In principle, potassium and oxygen vacancies (V_K_ and V_O_) should form and either remain in the near-surface or migrate into the bulk, promoting the migration of KO from the interlayers toward the surface. DFT calculations and photoelectron spectroscopy data below provide supporting evidence for these processes.

### DFT Calculations

**Figure 5:**
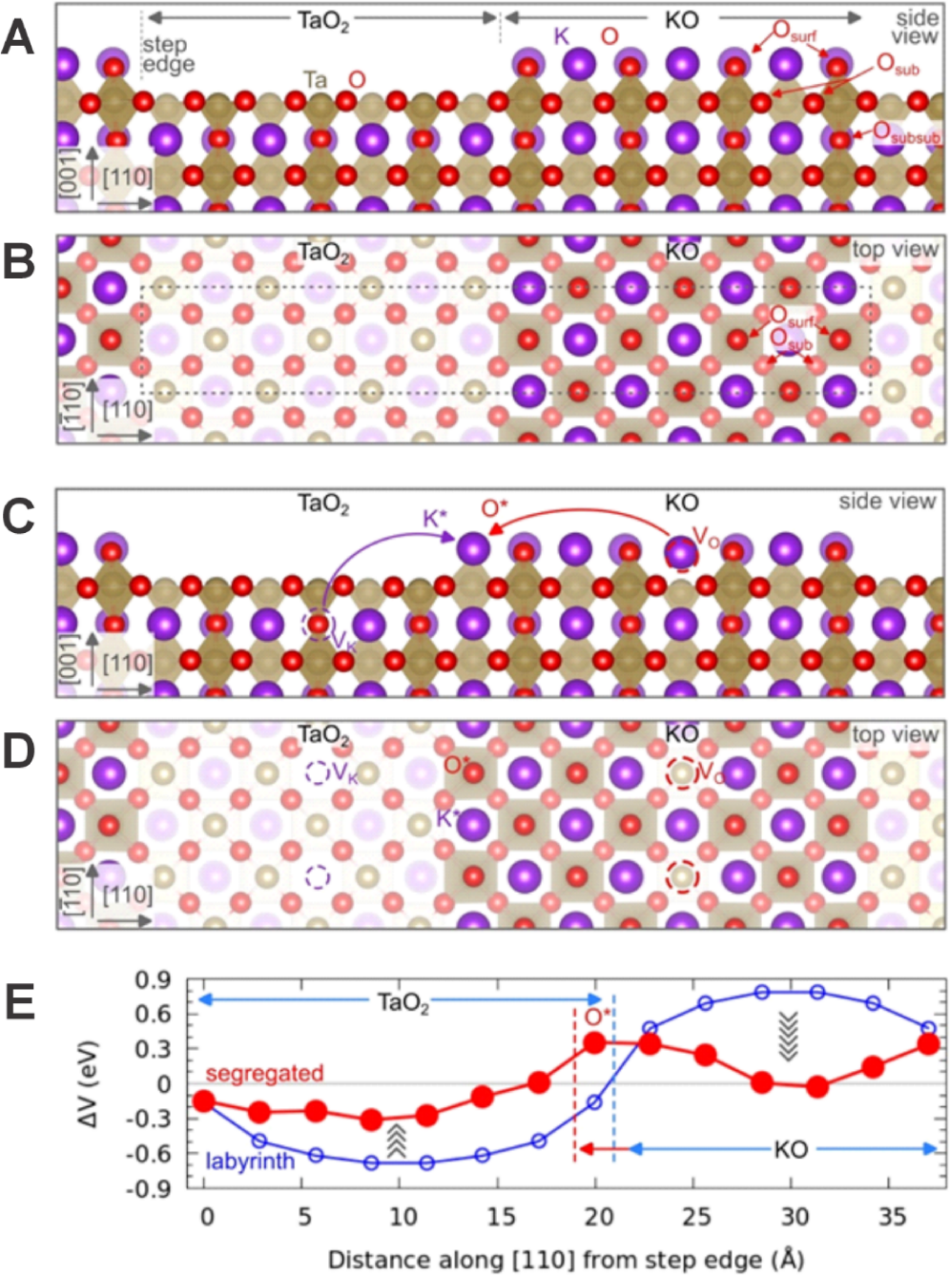
DFT modeling of KO segregation. (A, B) Side and top view of the unreconstructed labyrinth phase with a slab size of . The labels indicate the TaO_2_ and KO terraces, as well as the oxygen atoms on the surface layer (O_surf_), and on subsurface layers (O_sub_ and O_subsub_). The dashed rectangle in part B represents the unit cell used in the calculations. (C, D) Analogous model for the segregated phase (K* and O* atoms have been moved as indicated by the arrows in C). (E) Average electrostatic potential (Δ*V*) on oxygen atoms lying on the TaO_2_ termination and below the KO terrace. The *x* axis represents the distance of every O atom along [110] from the TaO_2_/KO step edge. The segregated phase (filled red circles) shows a smaller variation of Δ*V* as compared with the unreconstructed labyrinth (empty blue circles).

The feasibility of the KO segregation has been investigated by density functional theory (DFT) calculations, and the results are summarized in Figure 5 and Table 1. We modeled the labyrinth phase with KO/TaO_2_ stripes showing different lateral width (√2 × *n*√2 R45°, with *n* = 4, 5, 6, 7). KO segregation was studied by removing one oxygen and one potassium atom from their original sites and by placing them on lattice positions at the surface step edge, thus enlarging the KO terrace (see Figures 5A–D and S9). Generally, the KO segregation is driven by the uncompensated polarity of the (KO)^−^ and (TaO_2_)^+^ terminations.^14^ To act as a polarity-compensating species, the potassium vacancy V_K_ is located below the TaO_2_ termination, while the oxygen vacancy V_O_ is at or below the KO terrace.

**Table 1:** Energy Stability of the KO Segregation^a^

Cell size	Defect configuration	Energy (eV)
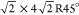	K, O (surf)	+1.31
	K, O (sub)	+1.14
	K, O (subsub)	+1.25
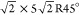	K, O (surf)	+0.51
	K, O (sub)	+0.96
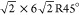	K, O (surf)	+0.25
	K, O (sub)	+0.74
	K, O (subsub)	+0.86
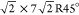	K, O (surf)	**–0.04**
	K, O (sub)	+0.43

a Most favorable configurations of potassium and oxygen vacancies in the segregated phase, shown here for different slab sizes (see Supporting Information for the complete set of data): V_K_ occupies central sites below the TaO_2_ termination, while V_O_ occupies central sites on the KO terrace (surf) or on the first (sub) and second subsurface layers (subsub).

Table 1 shows the energy stability of K and O vacancies formed at (or below) the center of the TaO_2_ and KO terraces, respectively: vacancies on these central sites result in better stability and more efficient compensation of surface polarity, as compared to vacancies closer to the terrace edges (see Table S3). Remarkably, the KO segregation shows a strong impact on the electrostatic properties of the surface. Figure 5E shows the polar electrostatic potential acting on surface oxygen atoms along [110], in both the unreconstructed and segregated phases (analogous results are shown in Figure S10 for the Ta and K sites). The site-dependent difference in the values of the potential is due to the polar electrostatic field, which shows opposite directions on the TaO_2_ and KO terminations, as well as a stronger effect on the central sites of the labyrinth stripes (see open blue circles in Figure 5E). As marked in the figure by the gray arrows, the variation of electrostatic potential along the TaO_2_ and KO terraces is sizably reduced by the K and O vacancy/segregation (filled red circles). Thus, the KO segregation improves the polarity compensation on the surface, as compared to the labyrinth phase. We recall that in the labyrinth phase, the variation of the polar electrostatic potential is already smaller as compared to large KO and TaO_2_ terraces^14^ on as-cleaved surfaces. Remarkably, KO segregation compensates the polarity to an even greater extent.

We note that the energy balance depends on the lateral size of the KO/TaO_2_ terraces. The process is slightly unfavorable for small terraces and turns favorable for the  slabs (Figure S11). This corresponds to a terrace width slightly larger than that observed experimentally (Figure 1C,D). The typical experimentally observed cell size corresponds to . This slight quantitative discrepancy with the experiment might be due to the ordered arrangement of vacancy defects in our models (in our computational setup, vacancies are constrained to align along the [−110] direction). While point defects might arrange themselves in certain configurations to minimize energy (forming the so-called extended defects),^21,22^ an exhaustive exploration of such a defect configuration space is computationally prohibitive due to the required slab sizes. The energies summarized in Table 1, however, clearly confirm the plausibility of the proposed mechanism. While the KO segregation becomes energetically favorable at the slab size of , the energy cost for smaller KO/TaO_2_ stripes is only a few tenths of eV. These values are rather small as compared to the defect formation energies in bulk and on the unreconstructed labyrinth surface (∼7 and ∼5 eV for O and K vacancies, respectively, see also Table S4).

### X-Ray Photoelectron Spectroscopy

**Figure 6:**
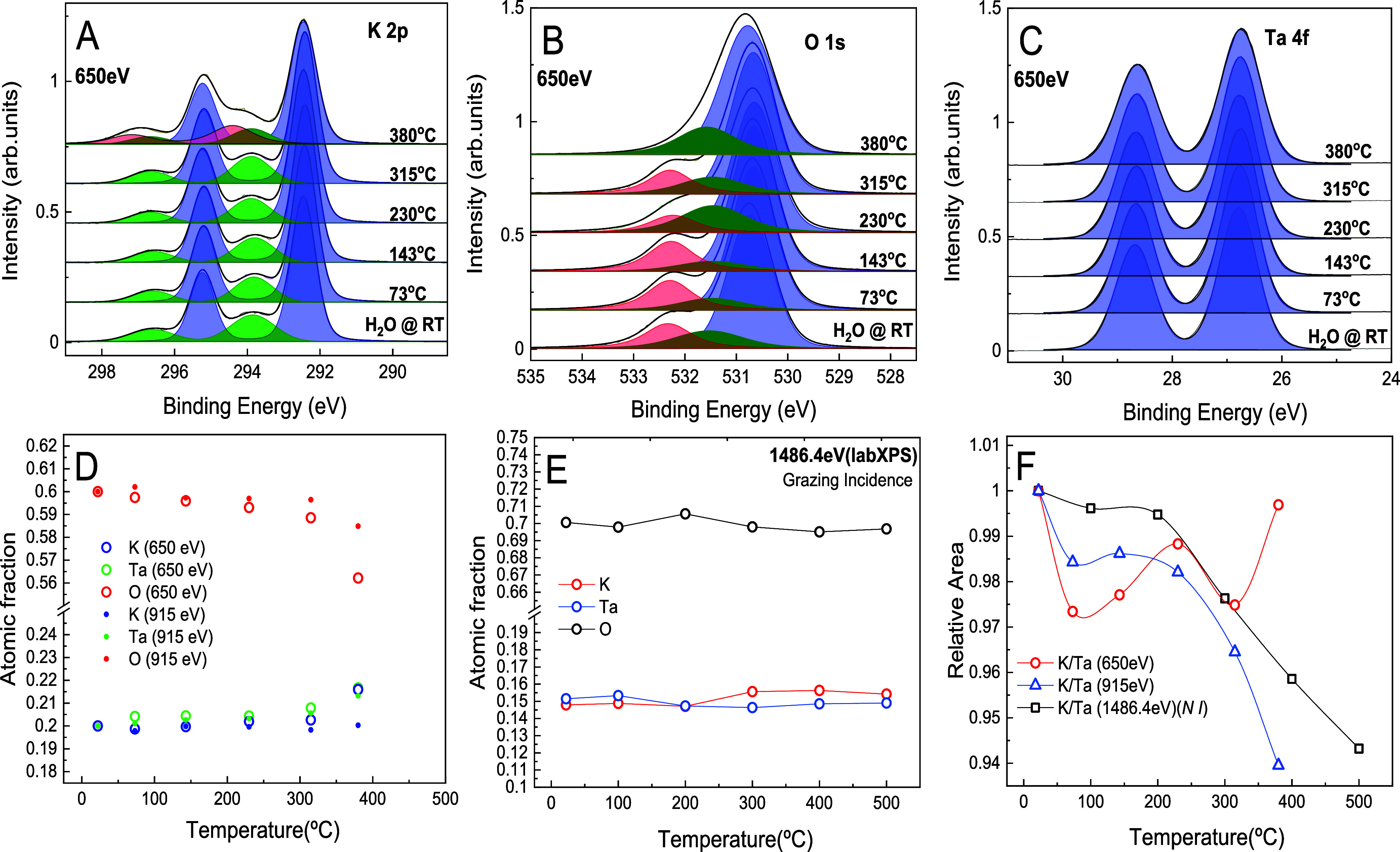
The evolution of PES signals recorded at the annealed KTaO_3_(001) surfaces. The first row shows the fitted spectra of (A) K 2p, (B) O 1s, and (C) Ta 4f regions. (D) Evolution of atomic concentrations determined from photoemission spectra obtained using two incident photon energies: 915 and 650 eV (with higher surface sensitivity), respectively. (E) Evolution of atomic concentrations determined from the take-off angle of 55°. (F) Evolution of K/Ta in cases of 650, 915, and 1486.4 eV (take-off angle of 90°) up to 500 °C.

X-ray photoelectron spectroscopy with both synchrotron radiation (SRPES) and lab source (XPS) were used to track the evolution of the chemical composition of the near-surface region after annealing to progressively higher temperatures. The as-cleaved KTaO_3_(001) surface was first exposed to water vapor at RT (22 °C) to create the (2 × 1) reconstruction (Figure 4A,B), followed by annealing up to 620 °C (Figure 1B–E and G–J). After water dosing and after each annealing step, an overview spectrum and photoelectron spectra (SRPES and XPS) of the K 2p, O 1s, and Ta 4f peaks were measured (Figures 6A–C, S4, S5 and S6). Photon energies of 650, 915, and 1486.4 eV were used to obtain data with different information depths (see Table S1 for details), and the quantification of the contribution of each element (K, Ta, O) is plotted in Figure 6D,E. The binding energies (BE) were calibrated so that the Ta 4f_7/2_ peak in the bulk KTaO_3_ sample equals to 26.7 eV.^54^ The elemental composition was evaluated from the corresponding peak areas, considering the electron inelastic mean free path and energy-dependent photoionization cross sections, and finally normalizing the data in a way that the initial composition matches the stoichiometry of KTaO_3_ (1:1:3). Uncorrected data are shown in Figure S3, together with a detailed description of the procedures, eqs S1, S2 and S3, and constants used (Tables S1 and S2).

The trends shown in Figure 6D are consistent with those of the model proposed in Figure 5. The potassium signal remains constant (within the experimental error) up to 315 °C and thereafter increases. Considering that the electron inelastic mean free path is 8.7 Å at a photon energy of 650 eV and 12.84 Å at a photon energy of 915 eV (see Table S1), this confirms the picture that the KO observed at the topmost surface layer does not diffuse from the bulk but originates from the exchange of surface ions with the subsurface layers (except for the final annealing). We estimate that interlayer exchange of ions should result in a change of XPS intensity within 1–2% (at the given mean free path), while diffusion over larger distances would change the signals by several percent). A clear trend in the annealing process is a gradual reduction in the oxygen content. This increases the atomic concentration ratio of both K/O and Ta/O in the PES data. The reduction of the O content is at least partially associated with the desorption of water from the surface and possibly also from the bulk; details are discussed in the Supporting Information.

Analysis of the peak shape of the single components shows that potassium (K 2p in Figure 6A) contains two components throughout the whole experimental procedure. The dominant K 2p_3/2_ peak is at 292.4 eV, indicating a +1 oxidation state for potassium, with an additional contribution at higher BE (293.8 eV) that might originate from the band-bending near the surface. After annealing to the highest temperature of around 380 °C, an extra doublet Voigt function toward higher binding energy centered at 294.4 eV is required to make the appropriate fit, indicating the presence of an extra component or a charge state for the potassium coordinated on the surface or associated with the oxygen removal scenario and formation of additional oxygen vacancies. The relative intensity of this component is higher under more surface-sensitive conditions (photon energy 650 eV vs 915 eV (see Figure S7A,E)), indicating its origin from the surface or near-surface region. Notably, this component appears at the same temperature as the “α” defects (Figure 1H).

The spectra of the O 1s (Figure 6B) were fitted by three components at 530.7 (blue), 531.5 (green), and 532.3 eV (red). The blue peak fit corresponds to the lattice oxygen, whereas the green peak fit might represent the oxygen anions coordinated with fewer cations and surface defects^55^ present at the surface. The component at higher BE (red) includes the contribution from surface hydroxyls up to 200 °C and other oxide components bound with the surface after water desorption. These two higher eV peaks reflect the polarity compensation that occurs after water adsorption and desorption. We note that a certain amount of hydrogen might be present in the subsurface layers of KTaO_3_, affecting the fine structure of the O 1s peak. Detailed evolution of the O 1s peaks with annealing temperatures and the compensation mechanism is explained in Figure S7D,H.

The presence of Ta^5+^ states bound to the surface as tantalate oxide (Ta_2_O_5_) is represented by the fitted Ta 4f spectra. This component, fixed at 26.7 eV throughout the experiment, indicates that tantalum remained in the Ta^5+^ oxidation state, as the fitted peak is symmetric, and no supporting evidence of Ta^4+^ or any other oxidation states.^56^ Notably, a slight broadening in the spectra at 380 °C indicates an increased variation in Ta coordination changes attributable to the vacancies and relaxation phenomena. The temperature-dependent evolution of the Ta 4f peak is shown in Figure 6C.

The point defects marked in Figures 1G,H and 4H are of intrinsic character, i.e., and originate from water desorption and reorganization of K, Ta, and O atoms, as there is no evidence of the presence of any other elements in the overview spectrum. Detailed description of their geometric structure is beyond the scope of this work, some structures were proposed in a theoretical study in ref.^57^. The dark spots that appear at KO terraces (“α” defects in Figure 1G) tend to adopt a (2 × 2) ordering, which is consistent with the picture of the cation-exchange reconstruction predicted theoretically.^57^ Here, switching K^+^ and Ta^5+^ ions between the layers provides a full polarity compensation within a (2 × 2) cell. The elongated defect chains that appear at the KO terraces after annealing above 620 °C (“β” defects in Figures 1J and 4H) show a certain level of disorder and likely introduce tantalum into the KO layer, as suggested by the increase of the Ta signal after annealing to higher temperatures (Figure 6D–F). The slight disagreement in stoichiometry between PES and AFM measurements can be correlated to the probing depths, as the AFM only probes the topmost surface layer, which is 2 Å thick. For SRPES, even the most sensitive data here have an information depth of 15 Å.

## Conclusion

We discussed the stability of the KTaO_3_(001) perovskite surface and the possibility of using the polarity compensation mechanisms to drive the out-of-plane segregation of the alkali species. Unlike previous approaches, we chose to investigate clean bulk-terminated (1 × 1) as-cleaved surfaces and use the interaction with water vapor for the initial polarity compensation. The initial homogeneity and stability provided by the (2 × 1) reconstruction of the hydroxylated surface is lost upon annealing and is supplemented by an interlayer switching of potassium from the subsurface to the top surface. This enrichment of K on the surface is accompanied by the shrinkage of TaO_2_ terraces and subsequent growth of the KO area. Annealing to temperatures above 300 °C initially leads to the formation of point defects, which later cluster into arrays of one-dimensional chains. The rearrangement of atoms within the surface layers has a significant effect on the chemical reactivity of the surface, as demonstrated by the adsorption of water and carbon monoxide. DFT methods confirm that the formation of subsurface K and surface oxygen vacancies are favorable, as well as the segregation of KO toward the surface.

Segregation of alkali metals is typically considered to have a delirious effect in applications. It could be possibly suppressed by introduction of other efficient polarity-compensating mechanisms, such as doping of the surface TaO_2_ layer by cations of suitable valencies and sizes; this strategy already proved to be beneficial for avoiding segregation driven by the cation size mismatch.^31,58^

The model system of the bulk-terminated perovskite used in this study aims to represent perovskites prepared by wet chemical techniques and operated at low to intermediate temperatures (below ∼500 °C); such surfaces are typically assumed to follow the (1 × 1) surface periodicity. Our results are less relevant for materials prepared by high-temperature annealing (above ∼900 °C),^6^ where oxygen and alkali cations can evaporate and surface polarity can be compensated by different mechanisms.^59^

## Supporting Information Available

Estimation of potassium oxide (KO) and tantalum oxide (TaO_2_) areas from AFM images; a schematic representation of the experimental pathway; elemental composition of the surface measured by SRPES; an overview XPS spectrum of the pristine polarity compensated KTaO_3_(001) surface recorded at a photon energy of 1000 eV; the evolution of XPS spectra (take-off angle of 55°); the evolution of XPS spectra (take-off angle of 90°); the evolution of synchrotron PES data and the percentage of oxygen components in O 1s peak throughout the experiment; large-scale topography of a cleaved crystal and determination of terminations; KO segregation; electrostatic potential; energy stability of the labyrinth phase; inelastic mean free path of photoelectrons in KTaO_3_ sample; theoretical photoionization cross sections of photoelectron peaks; energy stability of the KO segregation, with different distribution of atomic vacancies; defect formation energy (PDF)

## Notes

Original experimental data are available via Zenodo, DOI: https://doi.org/10.5281/zenodo.13986178 and RODBUK, DOI: https://doi.org/10.57903/UJ/VC1I41.
