# Supplementary material for: Atomic-Scale View at the Segregation of Alkali Metals toward the KTaO3(001) Perovskite Surface
Source: ACS Appl Mater Interfaces. 2024 Dec 10;16(50):70010–9. doi: 10.1021/acsami.4c13795 (PMC11660036; doi:10.1021/acsami.4c13795)
Supplement: Supplementary file 1 — am4c13795_si_001.pdf [file am4c13795_si_001.pdf]

Supporting Information

Atomic-Scale View at the Segregation of Alkali Metals toward the $KTaO_3$(001) Perovskite Surface

Aji Alexander,[1] Michele Reticcioli,[2**] Llorenç Albons,[1] Jesús Redondo,[1,3] Marco Corrias,[2] Igor Píš,[4] Zhichang Wang,[5] Viktor Johánek,[1] Josef Mysliveček,[1] Cesare Franchini,[2,6] Dominik Wrana,[1,7 *] Martin Setvin[1]

1. Department of Surface and Plasma Science, Charles University, 18000 Prague, Czech Republic
2. Faculty of Physics and Center for Computational Materials Science, University of Vienna, Sensengasse 8/12, 1090 Vienna, Austria
3. Department of Polymers and Advanced Materials, Centro de Física de Materiales, University of the Basque Country UPV/EHU, 20018 San Sebastián, Spain
4. CNR - Istituto Officina dei Materiali (IOM), 34149 Trieste, Italy
5. International Center for Quantum Materials, School of Physics, Peking University, 100871 Beijing, China
6. Department of Physics and Astronomy, Alma Mater Studiorum - Università di Bologna, 40126 Bologna, Italy
7. Marian Smoluchowski Institute of Physics, Jagiellonian University, 30-348 Krakow, Poland

* dominik.wrana@uj.edu.pl

** michele.reticcioli@univie.ac.at

Supplementary Figures and Tables (experimental part)

Figure S1: Estimation of Potassium oxide (KO) and Tantalum oxide (TaO_2) areas from AFM images.

Figure S2: A schematic representation of the experimental pathway.

Figure S3: Elemental composition of the surface measured by SRPES.

Figure S4: An overview XPS spectrum of the pristine polarity compensated $KTaO_3$(001) surface recorded at a photon energy of 1000eV.

Figure S5: The evolution of XPS spectra (take-off angle of 55°).

Figure S6: The evolution of XPS spectra (take-off angle of 90°).

Figure S7: The evolution of Synchrotron PES data and the percentage of oxygen components in O 1s peak throughout the experiment**.**

Figure S8: The surface of $KTaO_3$(001) after cleaving and the determination of KO and TaO_2 terminations based on AFM measurements

Table S1: Inelastic mean free path of photoelectrons in $KTaO_3$ sample

Table S2: Theoretical photoionization cross sections of photoelectron peaks

Supplementary Figures and Tables (theory part)

Figure S9: KO segregation.

Figure S10: Electrostatic potential.

Figure S11: Energy stability of the labyrinth phase.

Table S3: Energy stability of the KO segregation, with different distribution of atomic vacancies.

Table S4: Defect formation energy.

1. Proportion of KO to TaO_2 at the $KTaO_3(001)$ surface

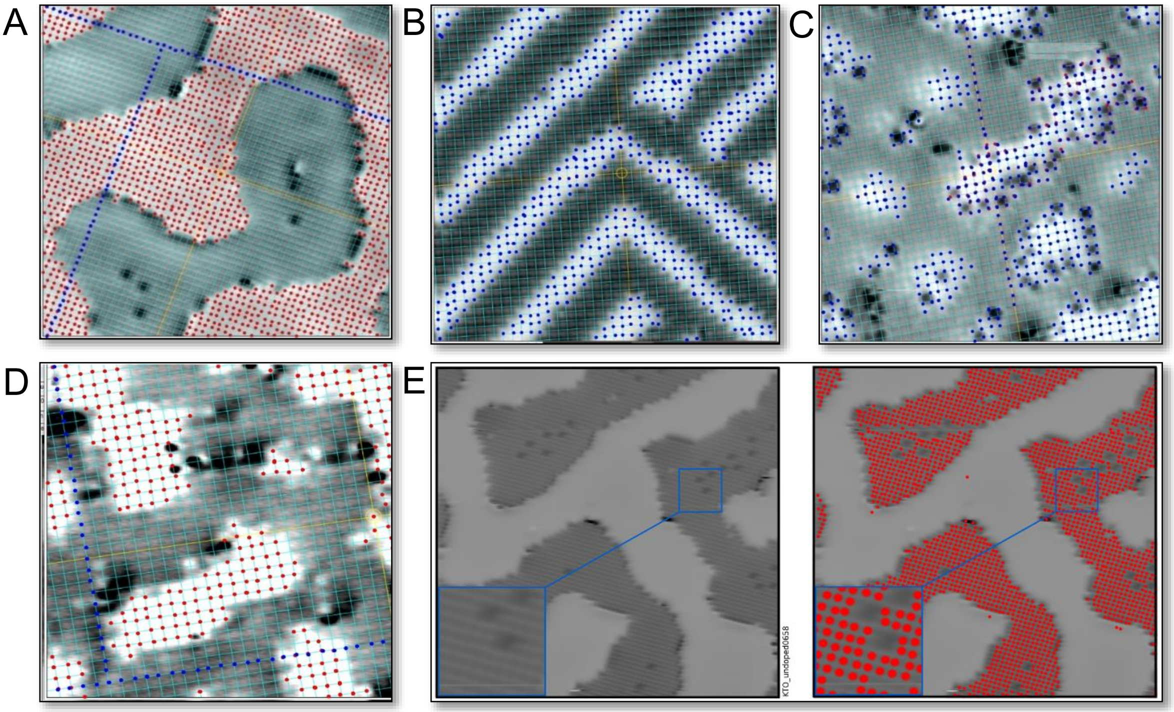

Figure S1: Estimation of potassium oxide (KO) and tantalum oxide (TaO_2) areas from AFM images. The ratio of KO : TaO_2 is A) 50.5:49.5 for the as-cleaved surface. B) 55:45 after annealing to 200ºC. C) 62:38 after annealing to 300ºC. D) 66:34 after annealing to 620ºC. E) The left panel shows a non-contact AFM image of the as-cleaved surface of $KTaO_3(001)$, counted using the AiSurf package.

The percentage of potassium oxide and tantalum oxide was estimated from the AFM images. The lattice grid was overlaid on the constant height AFM image (See Fig. D) and the number of K, Ta, and defects was counted manually. Another approach was using the Python package. Panel E shows the nc-AFM image of as-cleaved $KTaO_3(001)$, with and without atomic detection performed by AiSurf. The KO terminations are imaged as rows of dark dots corresponding to atoms. Raw image with zoomed image patch (left) and Image overlayed with red dots centered on the detected atomic positions(right). A total of 2233 atoms have been estimated.

2. Graphical Scheme

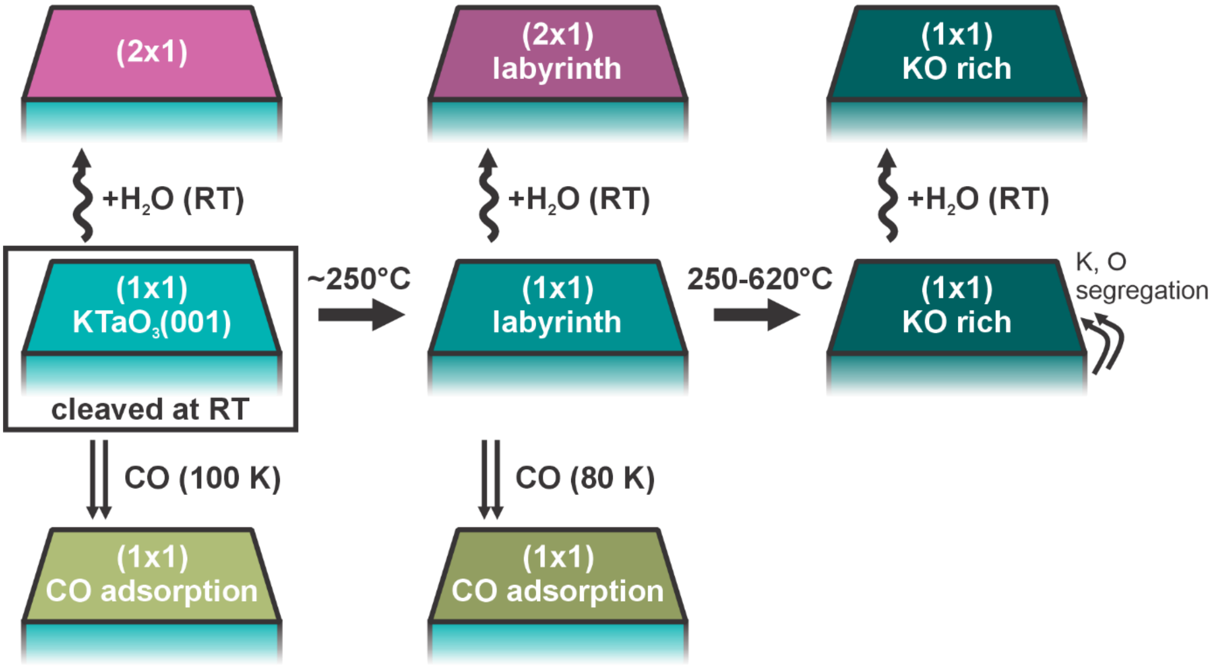

Figure S2: A schematic representation of the experimental pathway.

Fig. S2 summarizes our experimental approach to investigating the cation segregation at the surface of $KTaO_3$(001). To ensure the cleanest starting conditions we chose to cleave the potassium tantalate in situ at room temperature and perform measurements without breaking the vacuum. To assess the stability of the surface and track the KO enrichment at the surface (1x1) surface was annealed in UHV to temperatures up to 620 °C and at each step, the atomic structure of the surface as well as chemical composition were checked. The stability of the surface was probed by the *in-situ* exposure to water vapour, proving that surfaces annealed under 300 °C were still prone to the KO dissolution making the homogeneous (2x1) reconstruction. CO exposure to the surface was used as a final experimental confirmation of the KO/TaO_2 ratio evolution with annealing temperature and thus different reactivity of the $KTaO_3$(001).

3. XPS – inelastic mean free path of photoelectrons

Photon Energy	650 eV	915 eV	1486.4 eV
**Potassium (K 2p)**	8.77 Å	12.84 Å	21.03 Å
**Tantalum (Ta 4f)**	12.8 Å	16.64 Å	24.55 Å
**Oxygen (O 1s)**	5.0Å	9.26 Å	17.77 Å

Table S1: Inelastic mean free path of photoelectrons in $KTaO_3$ sample for different incident photon energies calculated using TPP-2M formula[1], assuming a density of 6.98g/cm3, a bandgap of 2.1eV, and 24 valence electrons per molecule.

4. Quantitative analysis with XPS

The chemical composition of the surface can be determined from the photoelectron peak intensities divided by the sensitivity factors. Sensitivity factors (SF) in XPS are empirically derived factors (from compounds of known composition) by which peak intensities are normalized (divided by) to provide the atomic concentration.

In the case of a flat, homogeneous, semi-infinite sample,

$$lx \propto Cx\ LMx\ Px\ T\ F \tag{S1}$$

,where:

- lx is the peak intensity extracted from the photoemission spectrum,
- Cx is the atomic density (number of atoms per unit volume),
- LMx is the electron inelastic mean free path,
- Px is the photoionization cross-section,
- T is the analyzer transmission function,
- F is the flux of incident X-rays.

Atomic sensitivity factor (SF) can be given by accounting for all the parameters of the specimen and spectrometer.

i.e.,
$$SF = LMx\ Px\ T = LMx \times \sigma_i \tag{S2}$$

where σ_i is the differential cross-section.

The atomic fraction of the element i in the homogenous sample with the total element of j can be expressed as:

$$Xi = (Ii/SFi)/(sum(\frac{Ij}{SFj}) \tag{S3}$$

The Relative Sensitivity Factors (RSF) for K 2p, O1s, and Ta 4f were determined from the spectra obtained after cleaving the sample, with a 1:1:3 (K: Ta: O) stoichiometry. The RSF values are 1.242 for K 2p, 2.690 for Ta 4f, and 0.3809 for O 1s.

The theoretically calculated sensitivity factors are, obtained by multiplying the differential cross section with the inelastic mean free path as given in Eq. 2, 0.959 for K 2p, 2.680 for Ta 4f, and 0.386 for O 1s. Using these sensitivity factors, the composition is determined to be 1.26:1:2.95 (K: Ta: O) stoichiometry, while relative changes in composition are determined with a precision of better than 1 atomic percent and an error in the intensities extracted from the photoemission spectra is less than 1%.

**hν = 650 eV**	**Total photoemission cross-section (σ_{tot})**	**Core-level asymmetry parameter (β)**	**Differential cross-section (σ_i)**
**K 2p**	0.343	2	0.772
**Ta 4f**	0.570	1.47	1.094
**O 1s**	1.360	0.86	2.094

**hν = 915 eV**	**Total photoemission cross-section (σ_{tot})**	**Core-level asymmetry parameter (β)**	**Differential cross-section (σ_i)**
**K 2p**	0.146	2	0.328
**Ta 4f**	0.222	1.44	0.421
**O 1s**	0.554	0.98	0.894

Table S2: Theoretical photoionization cross sections of photoelectron peaks for different photon energies[2].For linearly polarized light, the photoemission cross-section for photoelectron peak i is approximated by the differential cross section calculated for the angle of 30° between the polarization vector and photoemission direction ($\sigma_i = \sigma_{tot}(1+0.625\beta)$).

The errors in the theoretical parameters can be approximated as follows: cross-sections are around 10%, IMFP is about 15%, and the resulting determined stoichiometry has approximately 25% total error, which is systematic for data points of each specific elements[3]. Upon examination of the K2p, K2 s and

K 3p core-level intensities (Fig. S4) normalized to the theoretical sensitivity factors, we estimate that the absolute uncertainty may be as high as 47% (while relative uncertainty within one data set is still below 1%).

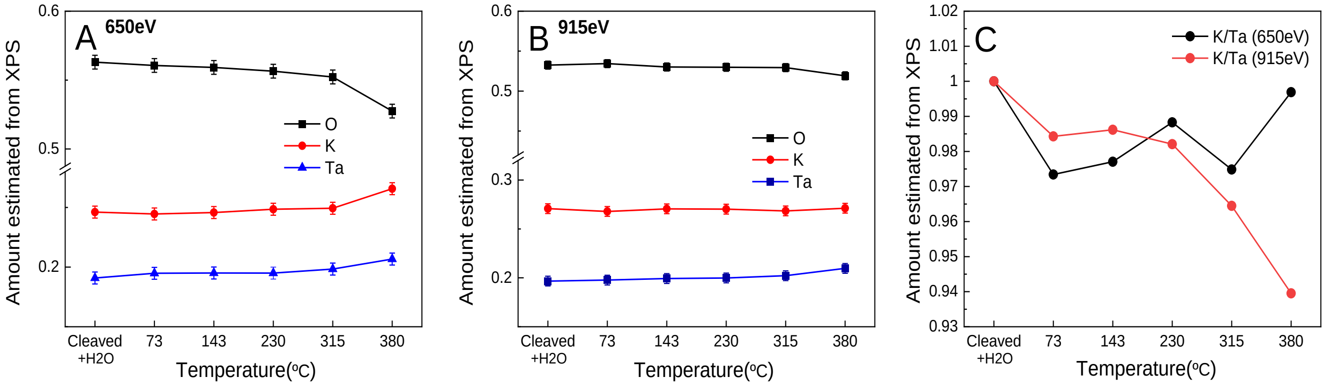

Figure S3: Elemental composition of the surface measured by SRPES after annealing to gradually increasing temperatures. A) Incident photon energy 650 eV, B) photon energy 915 eV. C) ratio between K and Ta evaluated from plots A, B.

Note: The photon flux was derived from the total photoelectric current recorded at the last mirror of the beamline. For the angular analyzer mode and small beam size, the transmission function can be regarded as constant with respect to photoelectron kinetic energy[4].

5. Photoelectron Spectroscopy - Additional data 1

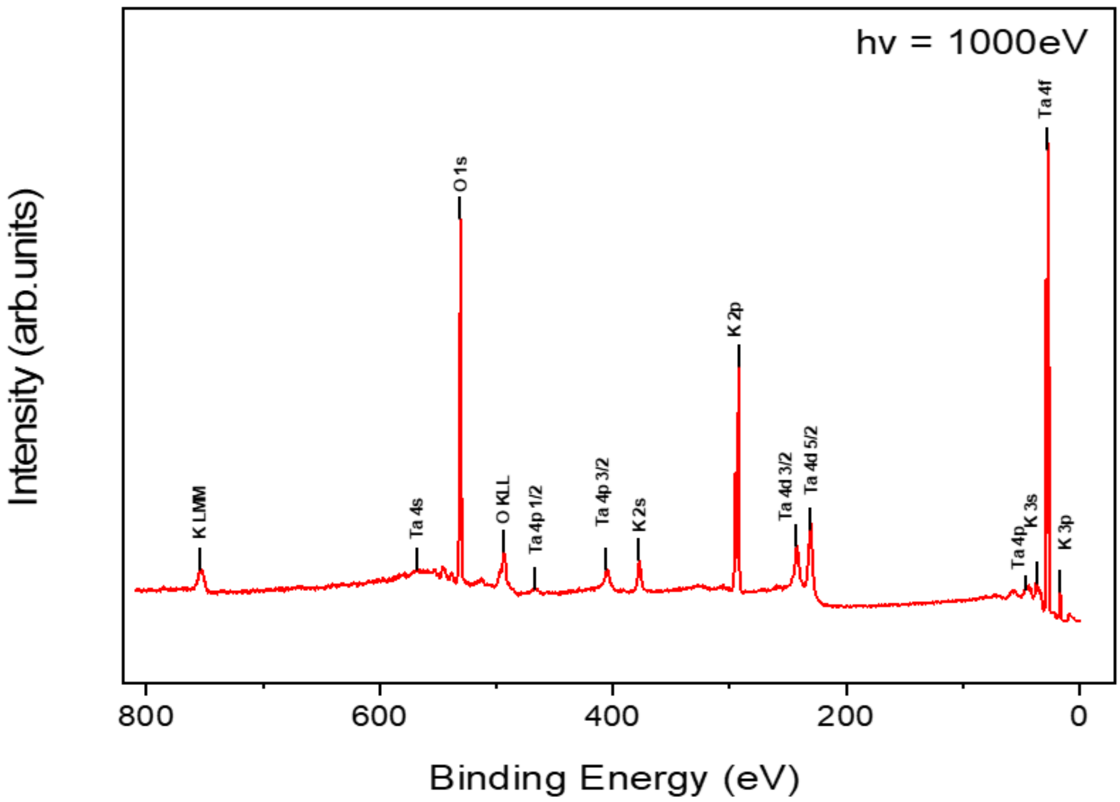

Figure S4: An overview XPS spectrum of the pristine polarity compensated $KTaO_3$(001) surface recorded at a photon energy of 1000eV, showing no detectable amounts of contaminants in the crystal.

6. Photoelectron Spectroscopy (Lab Source)- Additional data 2

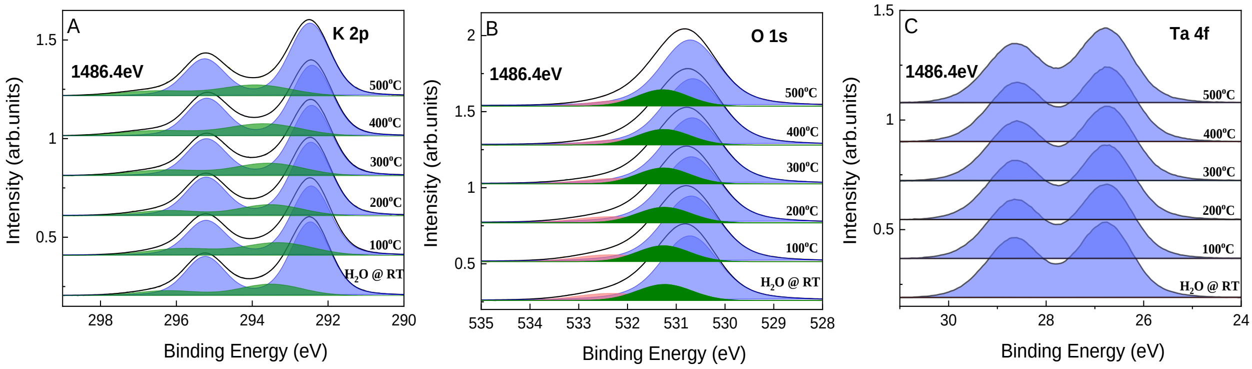

Figure S5: The evolution of XPS spectra of A) K 2p, B) O 1s, C) Ta 4f regions for the incident photon energy of **1486.4eV** at **take-off angle of 55°** (between RT and 500ºC).

7. Photoelectron Spectroscopy (Lab Source)- Additional data 3

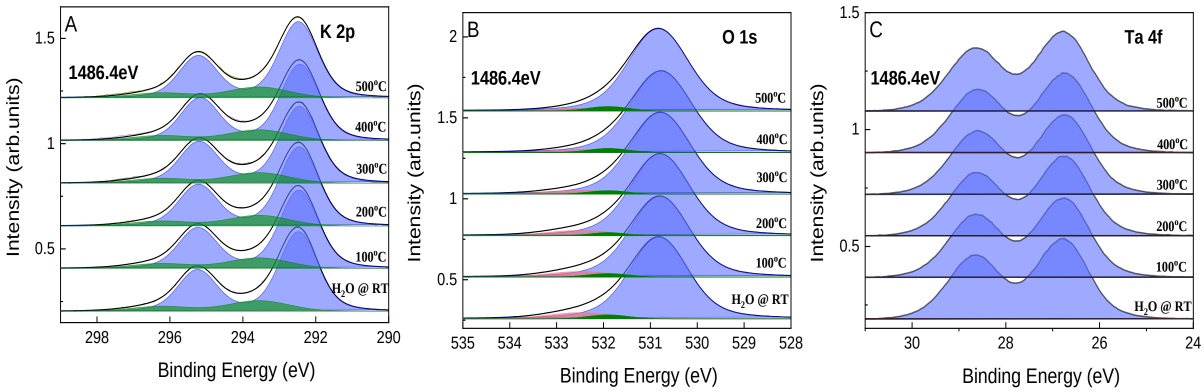

Figure S6: The evolution of XPS spectra of A) K 2p, B) O 1s, C) Ta 4f region for the incident photon energy of **1486.4eV** at **take-off angle of 90°** (between RT and 500ºC).

8. Photoelectron Spectroscopy - Additional data 4

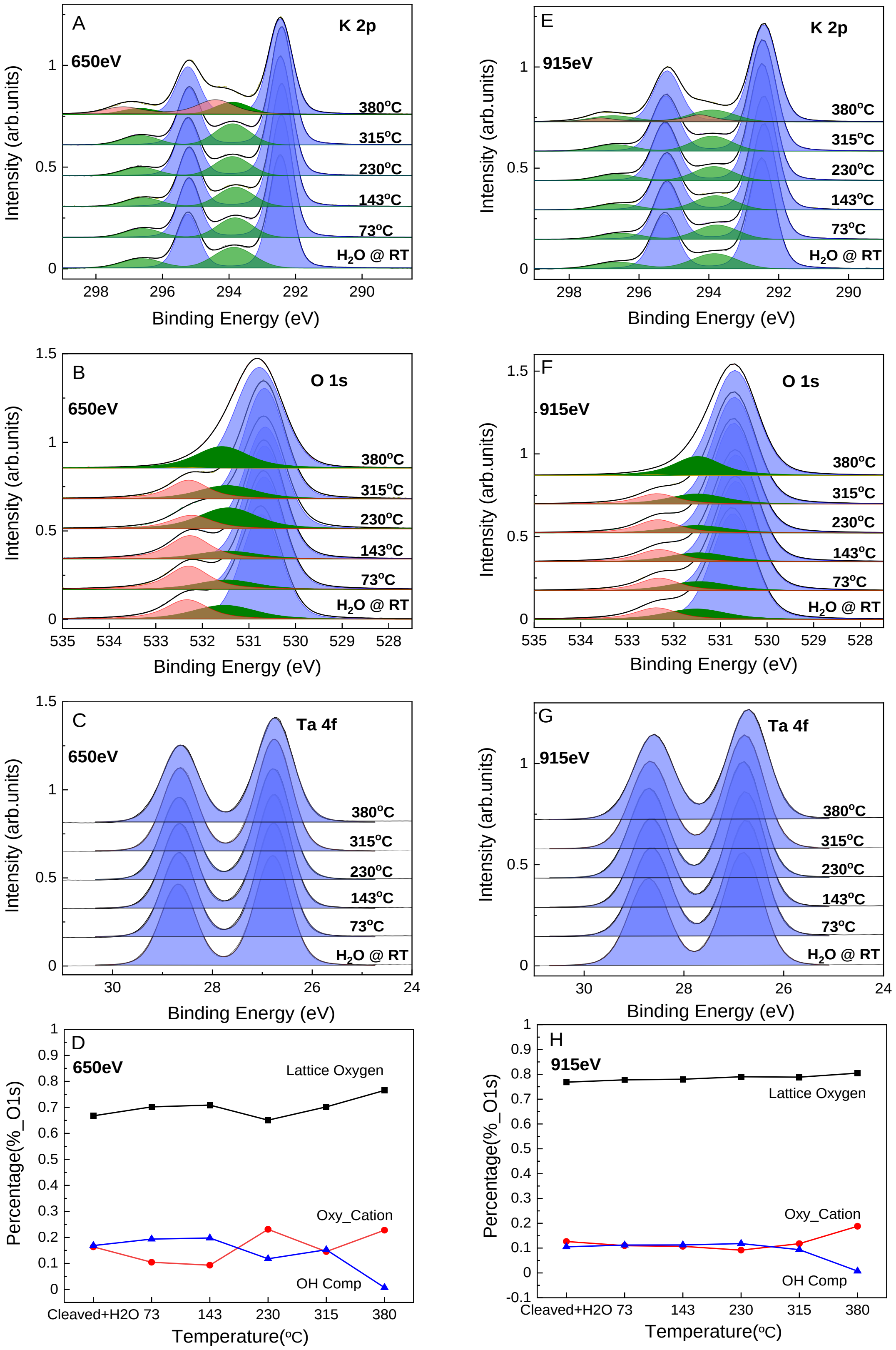

Figure S7: The evolution of the PES spectra at different temperatures for 650eV (left panels) and 915 eV (right panels). (A, E) K 2p, (B, F) O 1s, (C, G) Ta 4f, (D, H) Percentage of Oxygen components.

Fig. S7 shows all the XPS data measured with incident photon energies of 650 and 915 eV (panels A to C). The O1s peak has a complex structure, which shows a nontrivial evolution during the experiment, as summarized in panel D. Notably, the (2×1) reconstruction is known to be highly sensitive to irradiation with electrons[5] and X-rays. Consequently, the XPS spectra corresponding to the room-temperature data are likely influenced by this effect due to the high intensity of the used synchrotron beam. For cleaved $KTaO_3$ samples, the O1s component always shows a shoulder on the high binding energy site. This shoulder is fitted here by a Voigt function centred at 532.2eV. These peaks are more pronounced in the surface-sensitive data measured with 650 eV incident photon energy; therefore, they are related to the surface layers of atoms. The peak at 532.3eV is predominantly attributed to hydroxyls in the (2×1) reconstruction because this is known to desorb below 200°C, where this peak is significantly reduced. The other peak at 531.5eV is tentatively attributed to surface hydrogen atoms, which are known to easily diffuse in the $KTaO_3$ lattice,[6] or from surface oxygen atoms that contribute to compensating the surface polarity ($O^{-\delta}$).

9. Large-scale topography of a cleaved crystal and determination of terminations

$KTaO_3$ cleaving process in the vast majority of individual cases results in smooth areas of (1x1) reconstruction, spanning from single micrometers to hundreds of micrometers, separated with multisteps of tens to hundreds of nanometers. A typical appearance of a cleaved crystal is shown in Fig. S8 A, with an inset showcasing a large-scale SEM image of the resulting surface with flat areas of tens of micrometers.

As the perovskite crystal is cleaved in the (001) direction, with a layered structure of alternating KO/TaO_2 planes, two surface terminations have equal coverages after the cleaving. The KO/TaO3 terminations form stripes with a characteristic width in the range from 3 to 8 nm, which is a consequence of the polarity compensation mechanism of a polar surface, which is trying to limit the size of domains with uncompensated polarity.

Fig. S8 shows typical images of a cleaved $KTaO_3$(001)-(1x1) surface obtained by non-contact AFM, in two different modes of operation. When a surface is measured with a set constant frequency shift (Fig. S8B) and an additional Kelvin loop to account for electrostatic interactions. KO terraces protrude from the surface approximately half of a unit cell (about 1.9 Å), compared to the TaO_2 regions that are in the trenches. In the constant height mode (Fig. S8C), the interaction between the tip and the surface is stronger at KO stripes, hence the increased frequency shift and the visible atomic arrangement of the K cations (see Fig. 1 F-J in the main text). Fig. S8C also shows a typical constant height image of $KTaO_3$(001), together with the tunneling current acquired simultaneously. This constitutes direct proof for the valid KO/TaO_2 terminations assessment. As two terminations nominally carry an excess charge (KO^- and TaO_2^+), there are differences in the electronic structures of both, resulting in TaO_2 terraces being conductive and KO insulating.

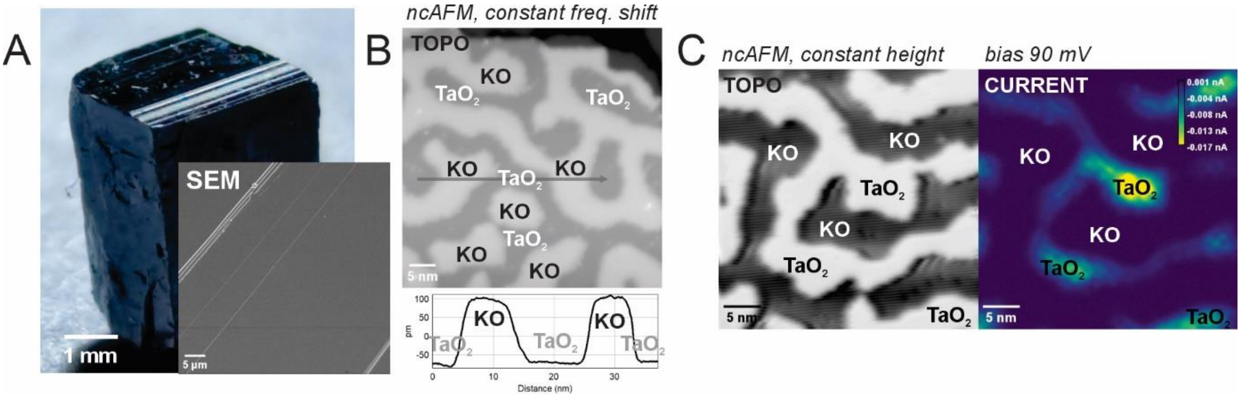

Figure S8: As-cleaved $KTaO_3$(001) surface and crystal. A) A photo of a cleaved Sr-doped crystal with an inset SEM image showing flat surface, B) Constant freq. shift imaging of $KTaO_3$(001)-(1x1), C) Constant height topography and current maps acquired at Sr-doped surface.

9. Additional data from DFT calculations

In this Section, we include additional data as obtained from our density functional theory calculations.

We modelled the KO segregation considering different sites for the K and O vacancies. Figure S8 shows the $\sqrt{2} \times 7\sqrt{2}R45°$ slab model, including the labeling for the atomic vacancies. The labeling is analogous for the smaller slabs, with the numbering starting from the right-hand side step edge (indicated as e0).

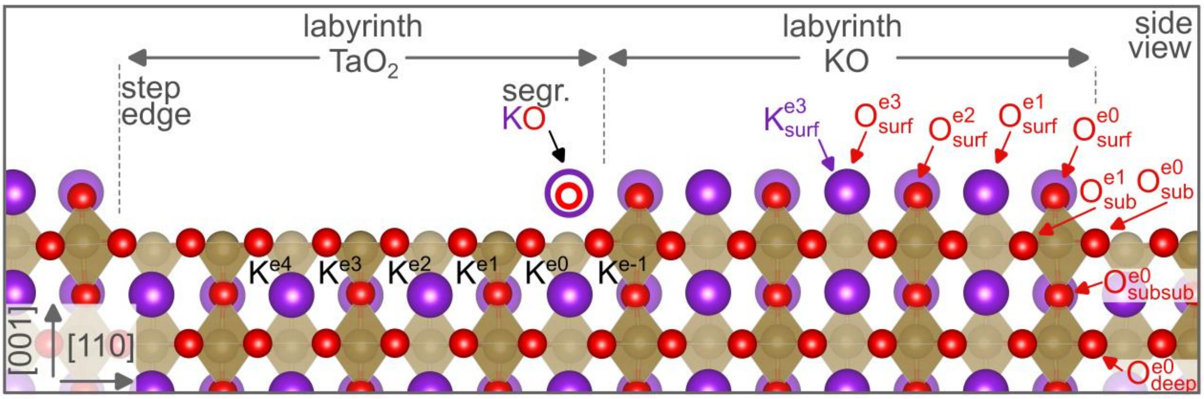

Figure S9: KO segregation. This model shows the labelling of the atomic sites considered for the formation of K and O vacancies. The counting starts from the right-hand side step edge (labelled as e0). The purple and red circles represent the sites of the segregated K and O atoms.

In Table S3 we report the energy stability of the segregated phases with different distributions of the atomic vacancies. As discussed in the main text, due to the polar field, vacancies forming at/below the centre of the TaO_2 and KO terminations show more favourable energy as compared to vacancies closer to the step edge. Moreover, we note that sites at or closer to the KO surface layer show better stability for the O vacancies as compared to sites on deeper layers (the precise trend depends on the size of the modelled TaO_2/KO stripes).

Importantly, particular arrangements of defects show energy stability slightly larger than the unreconstructed labyrinth phase; moreover, in the case of the $\sqrt{2} \times 7\sqrt{2}R45°$ slab, two vacancy configurations reported in the Table are energetically more favorable than the labyrinth structure (highligted in red). The most favorable configuration ($K^{e3}_{surf} – O^{e3}_{surf}$) includes both the K and O vacancies on the terminating KO terrace: Therefore, this solution represents an intermediate step in the process of forming thinner TaO_2/KO stripes, thus confirming the labyrinth with smaller stripes as lower temperature ground-state phase[1]. The formation of K vacancy below the TaO_2 termination ($K^{e3} – O^{e3}_{surf}$) rather than on the KO surface layer is only 70 meV less favourable in our computational setup. As discussed in the main text, we recall that the energy of segregated phases is likely to be artificially penalized by the small size of our models in the [-110] direction (i.e., the direction running along the step edges): While our setup constrains the vacancies to be aligned along [110], disordered arrangements could improve the energy stability.

Table S4 shows the defect formation energy of K and O vacancies in both the $KTaO_3$ bulk phase and on the unreconstructed labyrinth surface. The energy cost to form point defects (vacancies) ranges from ~5 to ~10 eV depending on the specific case considered.

Cell size	K vacancy	O vacancy	Energy (eV)
$\oplus \sqrt{2} \times 4\sqrt{2}R45°$	e2	surf e2	+1.31
	e2	sub e2	+1.14
	e2	subsub e2	+1.25
	e2	deep e2	+1.98

$\sqrt{2}\times 5\sqrt{2}R45°$	e2	surf e2	+0.51
	e2	sub e3	+0.96
	e2	sub e4	+1.02 (TBC)
$\sqrt{2}\times 6\sqrt{2}R45°$	e3	surf e3	+0.25
	e3	sub e3	+0.74
	e3	subsub e3	+0.86
$\sqrt{2}\times 7\sqrt{2}R45°$	e3	surf e3	**-0.04**
	surf e3	surf e3	**-0.11**
	e0	surf e0	+1.00
	e3	sub e4	+0.43
	e3	sub e3	+0.50
	e4	sub e3	+0.48
	e -1	sub e3	+1.66

Table S3: Energy stability of the KO segregation, with different distribution of atomic vacancies. The energy values are referenced to the corresponding labyrinth phase. (The defects were modeled on either one of or both the two termination sides of our slab: for the most relevant cases, we checked that the asymmetric/symmetric modeling of the defects on our slabs had no sizable impact on the resulting energy value.)

Cell size	K vacancy	O vacancy	Energy (eV)
surface $\oplus \sqrt{2} \times 4\sqrt{2}R45°$	none	surf e1	+6.34
	none	sub e2	+6.90
	e2	none	+5.09
	e2	sub e2	+9.07
	e0	sub e3	+9.13
bulk 3x3x3	none	yes	+6.94
	yes	none	+5.66
	yes	yes	9.91
bulk 3sq2x3sq2x4	none	yes	+6.93
	yes	none	+5.63
	yes	yes	+9.93
bulk 3sq2x3sq2x4	none	yes	+6.90
	yes	none	+5.59
	yes	yes	+9.88

Table S4: Defect formation energy. The defect formation energy is shown for surface and bulk cells, including one K or O vacancy as well as both K and O vacancies simultaneously.

Figure S8 complements the data shown in the main text for the electrostatic potential. The segregated phase shows analogous effects on any atomic species: The variance of the electrostatic potential is reduced as compared to the unreconstructed labyrinth phase (as discussed in the main text for the O atoms).

Figure S10 shows the energy stability of the unreconstructed labyrinth phase as a function of the width of the TaO_2/KO stripes. The curve shows a minimum for the $\oplus\sqrt{2}\times 4\sqrt{2}R45°$ slab, as the result of a trade-off between polar field compensation and step edge cost. These results are in good qualitative agreement with our initial work on the $KTaO_3$(001) reconstructions,[5]

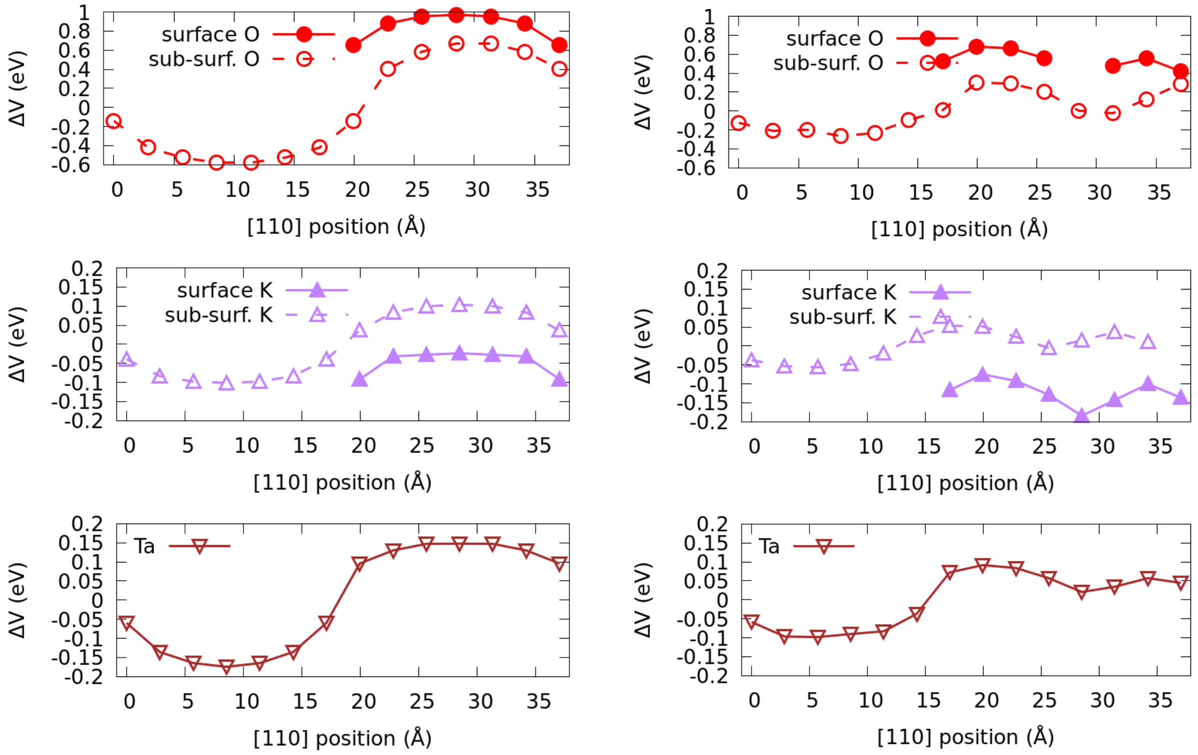

Figure S10: Electrostatic potential. The average electrostatic potential is shown for O and K atoms on the KO surface, and on the underlying TaO_2 layer (labeled as surface and sub-surf., respectively) together with the potential on Ta sites. Left panels refer to the unreconstructed $\sqrt{2}\times 7\sqrt{2}R45°$labyrinth phase, right panels to the (K^{e3} – O^{e3}_{surf}) segregated phase

obtained in a different computational framework (strongly constrained and appropriately normed meta-generalized gradient approximation, SCAN, with the inclusion of an on-site effective U of 4.0 eV on the d orbitals of Ta atoms, rather than the generalized gradient approximation, GGA, adopted in the original work). We underline the importance of a good sampling of the reciprocal space, in order to guarantee the convergence of the results (in both the SCAN and GGA setups): due to the relatively small size of the slab along [-110], a fine sampling along this direction is required.

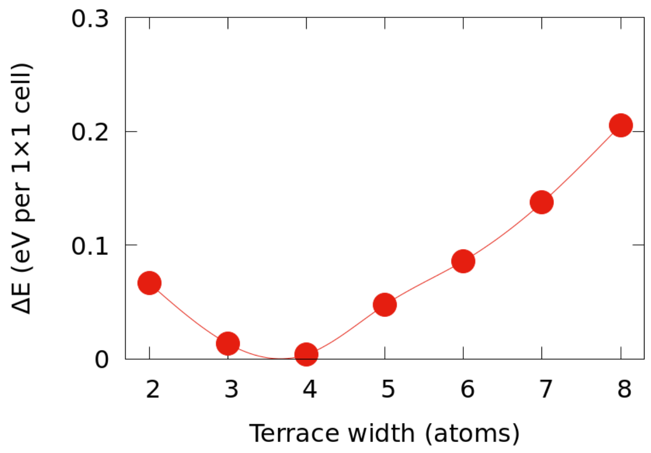

Figure S11: Energy stability of the labyrinth phase, with different size of the TaO_2/KO stripes (√2×n√2 R45º, with n=2, 3, 4, 5, 6, 7, 8).

References

(1) Tanuma, S.; Powell, C. J.; Penn, D. R. Calculations of Electron Inelastic Mean Free Paths (Imfps) .4. Evaluation of Calculated Imfps and of the Predictive Imfp Formula Tpp-2 for Electron Energies between 50 and 2000 Ev. *Surf Interface Anal* **1993**, *20* (1), 77-89. DOI: 10.1002/sia.740200112.

(2) Yeh, J. J.; Lindau, I. Atomic Subshell Photoionization Cross-Sections and Asymmetry Parameters - 1 Less-Than-or-Equal-to Z Less-Than-or-Equal-to 103. *Atom Data Nucl Data* **1985**, *32* (1), 1-155. DOI: 10.1016/0092-640x(85)90016-6.

(3) Shard, A. G. Practical guides for X-ray Photoelectron Spectroscopy: Quantitative XPS. *J Vac Sci Technol A* **2020**, *38* (4). DOI: 10.1116/1.5141395.

(4) Drera, G.; Salvinelli, G.; Åhlund, J.; Karlsson, P. G.; Wannberg, B.; Magnano, E.; Nappini, S.; Sangaletti, L. Transmission Function Calibration of an Angular Resolved Analyzer for X-ray Photoemission Spectroscopy: Theory vs Experiment. *J Electron Spectrosc* **2014**, *195*, 109-116. DOI: 10.1016/j.elspec.2014.06.010.

(5) Setvin, M.; Reticcioli, M.; Poelzleitner, F.; Hulva, J.; Schmid, M.; Boatner, L. A.; Franchini, C.; Diebold, U. Polarity Compensation Mechanisms on the Perovskite Surface $KTaO_3$(001). *Science* **2018**, *359*, 572-575. DOI: 10.1126/science.aar2287.

(6) Engstrom, H.; Bates, J. B.; Boatner, L. A. Infrared Spectra of Hydrogen Isotopes in Potassium Tantalate. *J. Chem. Phys.* **1980**, *73*, 1073. DOI: 10.1063/1.440279
